# Determination of heavy metals contamination in thyme products by inductively coupled plasma mass spectrometry

**DOI:** 10.1016/j.toxrep.2022.10.014

**Published:** 2022-10-27

**Authors:** Elias Akoury, Caline Baroud, Sally El Kantar, Hussein Hassan, Layal Karam

**Affiliations:** a Department of Natural Sciences, School of Arts and Sciences, Lebanese American University, Beirut 1102-2801, Lebanon; b Department of Nursing and Health Sciences, Faculty of Nursing and Health Sciences, Notre Dame University-Louaize, Zouk Mikael, Lebanon; c Nutrition Program, Department of Natural Sciences, School of Arts and Sciences, Lebanese American University, Beirut 1102-2801, Lebanon; d Human Nutrition Department, College of Health Sciences, QU Health, Qatar University, P.O. Box 2713, Doha, Qatar

**Keywords:** Toxic metals, Herbs and spices, Chemical contamination, Arsenic, Mercury, Lead, Cadmium

## Abstract

Thyme herbs constitute a major part of the Mediterranean diet and are gaining worldwide popularity. However, their chemical contamination with toxic metals may put consumers at a health risk. The objective of this study was to assess the incidence of Arsenic (As), Cadmium (Cd), Lead (Pb) and Mercury (Hg) in thyme-containing products. Composite samples were collected twice at six-month interval. Samples were digested by microwave digestion oven and analyzed by Inductively Coupled Plasma Mass Spectrometry (ICP-MS). 11%, 22%, and 86% of samples had unacceptable levels of As, Hg and Pb respectively according to the international standards set by Codex Alimentarius and all the samples had acceptable limits of Cd. This study highlighted the importance of monitoring and enforcing regulatory actions related to the contamination of the food chain with heavy metals.

## Highlights

• 36 composite samples of thyme herbs and thyme-based products were tested by ICP-MS.
• All the samples had acceptable limits of Cd according to Codex Alimentarius.
• 11%, 22%, and 86% of samples had unacceptable levels of As, Hg and Pb, respectively.

## 1 Introduction

*Thymus vulgaris*, commonly known as garden thyme, is an edible herb from the family of *Lamiaceae* grown in the Mediterranean region. The consumption of thyme in the Levant is a popular practice and an essential constituent in various dish recipes such as salads, thyme pizza known as Manooushe, thyme croissant, and others [1]. The main essential oils in thyme, Thymol and Carvacrol, have numerous health benefits such as antioxidant, anti-inflammatory properties, neurological and gastrointestinal effects [2]. Like many agricultural crops, the contamination of thyme by heavy metals originates mainly from polluted irrigation and contaminated soil. Heavy metals such as arsenic (As), cadmium (Cd), mercury (Hg), and lead (Pb) exhibit serious health concerns since they are non-biodegradable, have long biological half-lives, accumulate in the body and cause adverse effects. Ingestion of contaminated food and beverages is considered one of the major routes of exposure to these toxic metals [3]. Therefore, dietary exposure is an important step to assess the risk of heavy metals from thyme and its quantification of the likely intake is practiced through the dietary exposure assessment by combining food consumption data with the concentration of heavy metals [4], [5].

Many studies have been conducted to evaluate heavy metal contamination in herbs and spices as well as in other food products [6], [7], [8], [9], [10], [11]. However, no studies have been made to evaluate the occurrence of heavy metals in thyme and thyme products. The aim of this study was to assess the accumulation of As, Cd, Hg and Pb in thyme.

## 2 Materials and methods

### 2.1 Chemicals and reagents

Reagents used in this study were of high purity and high analytical grade: arsenic, cadmium, mercury, and lead standard solutions (Merck, Darmstadt, Germany), Hydrogen peroxide solution 30% (Sigma Aldrich, Germany), Nitric acid 69% (BDH Laboratory supplies, England), Hydrochloric acid 37% (AnalaR Normapur, France). The certified reference material for rice flour (IRMM-804) and for ground water (CRM BCR-610) were obtained from the Institute for Reference Materials and Measurements (Geel, Belgium) to validate the accuracy of the method. All solutions were prepared with analytical reagent grade chemicals and ultrapure water (18 MV.cm) obtained by purifying distilled water using a Milli-Q water purification system (Millipore S.A., St Quentin-en-Yvelines, France).

### 2.2 Collection of foodstuff and sample preparation

Thyme-based food products were selected (18 items), and a composite sampling approach was applied. This consisted of purchasing the same food item from five different brands/varieties found in representative retail markets in Lebanon. The samples were combined and blended to give a homogeneous composite sample which is representative of each food item. The complete set of samples was collected twice at six months’ intervals to take into account the effect of seasonal variation or difference in production dates on contamination levels. All items were tested as collected and were not subject to any cooking or preparation except pasta, pizza and tea (infusion and soaked). Pasta and pizza were prepared following the instructions of a traditional cookbook [12] using tomato thyme sauces and dried thyme as a seasoning herb. 0.75 g of tea was prepared in 100 mL as infusion by soaking the herbs in hot water or boiling the herbs with water. All samples were stored at − 18°C until analysis.

### 2.3 Sample digestion

Samples were digested using a Multiwave ECO microwave digestion system (Anton Paar GmbH, Graz, Austria) equipped with a rotor for 16 vessels after adding 8 mL of 69% nitric acid and 2 mL of 30% hydrogen peroxide to each of the samples (0.5 g). The sample digestion procedure was performed according to Table 1: (1) 850 W at 180°C for 10 min and (2) 850 W at 22 °C for 1 min for cooling. After microwave digestion, 2 mL HCl were added to sample solutions and the digested samples were then transferred into 50 mL flasks. The contents were diluted 5 times with 3% nitric acid prepared with ultrapure deionized water and were stored at 4°C until ICP-MS analysis.

**Table 1:** Operating Conditions for Microwave Oven Digestion.

Step	Power (W)	Pressure (MPa)	Temperature (^0^C)	Ramp time (min)	Hold time (min)
1	850	2	180	20	10
2	850	2	22	10	1

### 2.4 ICP-MS analysis

Elemental analysis was performed using inductively coupled mass spectrometer (ICP-MS) iCAP Q / iCAP RQ ICP-MS (Thermo Fisher Scientific Inc., Bremen, Germany) operating with argon gas of spectral purity (99.9995%). Before each experiment, the instrument was tuned using iCAP Q/RQ TUNE aqueous multi-element standard solution in 2% HNO_3_ + 0.5% HCl solution (Thermo scientific, Bremen, Germany). The torch position, ion lenses, gas output, resolution axis (10% of peak height) and background (<20 shots) were optimized with the tuning solution (1 mg.L^−1^) to carry out a short-term stability test of the instrument, to maximize ion signals and to minimize interference effects due to high oxide levels. The optimal parameters are shown in Table 2. Each solution was measured 3 times and the quantification of the heavy metals was carried out using external calibration curves. Standard solutions were prepared in 3% nitric acid, and for each element, the external calibration curve was plotted using eight different concentrations (Figure S1). The correlation coefficients for all the calibration curves were at least 0.9999, reflecting good linear relationship throughout the ranges of concentrations under study. All measurements were conducted using the full quantitative mode analysis while measuring several isotopes of the elements and checking the isotopic ratio in the digested samples to confirm the absence of polyatomic interferences. We used the most abundant isotope of every heavy metal (75As, 114 Cd, 202Hg and 208Pb) during the analysis of the results. The certified reference material was used to determine the accuracy of the method. Limit of detection (LOD) values for each element was determined by blank determination assays as 3 times standard deviation of 20 blank replicates. Limit of quantification (LOQ) values were calculated as 2 times LOD for each element. The precision for the analysis of heavy metals by ICP-MS was verified by applying the spiking methodology at different concentrations with permissible limits for each matrix; [13] and calculating the recovery percentages for each metal [14]. Each sample was collected twice (first collection and second collection), its heavy metal content was measured in three replicates and the average values are reported.

**Table 2:** Operating Conditions and Acquisition Parameters for ICP-MS.

Operating Conditions	
Spectrometer	Thermo Scientific, iCAP RQ, ASX-280, autosampler ICP-MS
Nebulizer	Borosilicate glass concentric with 0.4 mL/min
Spray chamber	2.70 °C, Quartz cyclonic
Cell geometry	octopole
Sampling cone	Nickel, 1.1 mm diameter orifice
Skimmer cone	Nickel, 0.75 mm diameter orifice
RF power	400 – 1600 W
Reflected power	< 10 W
Standard Mode	
Plasma gas flow	15 L/min
Nebulizer gas flow	1.03 L/min
Auxiliary gas flow	0.81 L/min
Expansion stage	2.01 mbar
Intermediate stage	10^–4^ mbar
Analyzer stage	10^–6^ mbar
He mode (collision cell mode)	
He gas flow	4.0 mL/min
Octopole bias (CCT bias)	– 21 V
Quadrupole bias (pole bias)	– 18 V
Acquisition Parameters	
Field	Virtual hyperbolic
Frequency	2 MHz
Mass range	2 – 290 a.m.u
Dwell time	0.04 s
Number of sweeps	5
Number of replicates	3
Total Acquisition time	220 s

## 3 Results and discussion

**Fig. 1:**
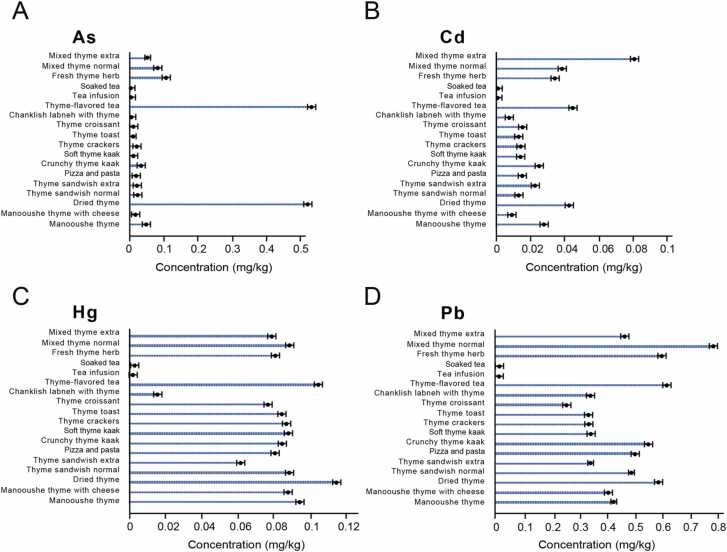
Mean Levels of (A) As, (B) Cd, (C) Hg, and (D) Pb in food (in mg/kg fresh weight) as represented in the bar diagrams.

In this study, we investigated the occurrence of As, Cd, Hg and Pb in 18 different individual foodstuffs using microwave-assisted digestion with ICP-MS elemental analysis. The range of concentrations of the 4 heavy metals obtained for each type of sample is shown in Table 3. The proficiency of the method was estimated by determining the limits of detection (LOD) and limits of quantification (LOQ) of every element studied [15]. The values of LOD and LOQ were in the range of 0.031–0.054 (ng/g) and 0.062–0.108 (ng/g), respectively. To check the accuracy of the method, two certified reference materials (ground water CRM BCR-610 and rice flour IRMM-804) were analyzed for the determination of As, Cd, Hg and Pb. The recovery percentages of these elements shown in Table 4 are within the interval of confidence (p < 0.05) calculated for the value certified. The analytical quality control was also confirmed by recovery measurements for the four elements on all thyme samples, spiking at two selected concentration levels, 200 and 1000 ng/g. The recoveries, depicted in Table 3 were in the range of 70–118% thus confirming that no significant element loss occurred during microwave digestion. Fig. 1 summarizes the results reported in Tables 3 and S1 reports the maximum allowed limits for As, Cd, Hg and Pb in foodstuffs according to local and international standards. For As, results showed that 89% (32/36) of the composite samples tested were acceptable, while 11% (4/36) were above the acceptable limits according to both national and international standards (Table S2). All samples examined for As were between the normative limits (0.2 mg/kg) except dried thyme (0.521–0.593 mg/kg) and thyme-flavored tea (0.534–0.684 mg/kg). Recent studies implicated high levels of As in thyme tea in Italy (0.33 mg/kg) and Tunisia (0.15 mg/kg); [16] in dried thyme (0.31 mg/kg) and oregano (0.37 mg/kg) in Latvian markets [17].

**Table 3:** Mean Levels of As, Cd, Hg, and Pb in food in mg/kg fresh weight.

	**As**		**Cd**		**Hg**					**Pb**		
	LOD	LOQ	LOD	LOQ	LOD		LOQ		LOD			LOQ
	0.000054	0.000108	0.000031	0.000062	0.000038		0.000076		0.000038			0.000075
**Foodstuffs**	**Average**	**Recovery %**	**Average**	**Recovery %**		**Average**		**Recovery %**	**Average**		**Recovery %**	
Manooushe thyme	0.031 ± 0.004	89.2	0.023 ± 0.002	103.5		0.092 ± 0.002		97.9	0.459 ± 0.003		94.6	
Manooushe thyme with cheese	0.012 ± 0.003	93.7	0.012 ± 0.001	73.9		0.087 ± 0.001		90.1	0.408 ± 0.004		74.2	
Dried thyme	0.557 ± 0.002	89.8	0.055 ± 0.003	81.4		0.119 ± 0.003		89.9	0.581 ± 0.003		77.1	
Thyme sandwich normal	0.022 ± 0.004	81.4	0.014 ± 0.001	82.9		0.087 ± 0.002		99.1	0.485 ± 0.004		70.4	
Thyme sandwich extra	0.016 ± 0.005	78.1	0.022 ± 0.002	87.4		0.073 ± 0.002		89.3	0.401 ± 0.005		90.2	
Pizza and pasta	0.015 ± 0.002	100.4	0.015 ± 0.002	100.1		0.079 ± 0.001		96.3	0.447 ± 0.002		94.4	
Crunchy thyme kaak	0.021 ± 0.003	78.4	0.022 ± 0.001	93.9		0.079 ± 0.004		102.2	0.511 ± 0.003		88.3	
Soft thyme kaak	0.008 ± 0.001	83.9	0.015 ± 0.003	73.7		0.084 ± 0.003		70.2	0.364 ± 0.004		80.8	
Thyme crackers	0.019 ± 0.002	87.7	0.016 ± 0.002	77.4		0.085 ± 0.002		72.4	0.374 ± 0.004		76.7	
Thyme toast	0.008 ± 0.001	98.9	0.015 ± 0.002	86.2		0.086 ± 0.003		111.5	0.189 ± 0.002		79.5	
Thyme croissant	0.009 ± 0.003	81.1	0.034 ± 0.004	109.6		0.079 ± 0.004		76.5	0.406 ± 0.002		96.4	
Chanklish labneh with thyme	0.007 ± 0.002	95.5	0.009 ± 0.001	114.2		0.047 ± 0.004		97.5	0.422 ± 0.001		75.5	
Thyme-flavored tea	0.609 ± 0.007	80.8	0.065 ± 0.005	82.3		0.107 ± 0.002		109.2	0.695 ± 0.006		90.2	
Tea infusion	0.001 ± 0.001	81.1	0.002 ± 0.001	118.5		0.003 ± 0.001		103.2	0.014 ± 0.001		101.8	
Soaked tea	0.001 ± 0.001	102.8	0.001 ± 0.001	86.4		0.004 ± 0.001		110.8	0.027 ± 0.002		89.7	
Fresh thyme herb	0.082 ± 0.004	89.4	0.029 ± 0.001	90.3		0.082 ± 0.003		92.3	0.718 ± 0.003		95.3	
Mixed thyme normal	0.091 ± 0.003	92.4	0.041 ± 0.003	100.4		0.091 ± 0.003		97.5	0.834 ± 0.004		93.5	
Mixed thyme extra	0.063 ± 0.003	98.8	0.088 ± 0.005	89.9		0.088 ± 0.002		102.3	0.498 ± 0.002		91.8	

LOD: limit of detection LOQ: limit of quantification. As and Pb spiked at 1000 ug/kg, Cd and Hg spiked at 200 ug/kg

**Table 4:** Heavy metals in certified reference materials.

Water reference material		
Heavy Metal	**Limit (mg/kg)**	**Recovery %**
As	0.01	105.1
Cd	0.003	108.3
Hg	n/a	n/a
Pb	0.008	120.6
Rice flour reference material		
Heavy Metal	**Limit (mg/kg)**	**Recovery %**
As	0.049	98.6
Cd	1.61	101.4
Hg	n/a	n/a
Pb	0.42	109.4

The analysis for Cd showed that all samples were below the permissible limit of 0.2 mg/kg with average concentrations ranging between 0.001 and 0.087 mg/kg. Among all, mixed thyme extra in the dried herb category had the highest concentration (0.0875 mg/kg) while soaked tea had the lowest level (0.01 mg/kg). A Total Diet Study (TDS) done on milk and dairy products in Spain found a mean concentration of Cd at 0.0085 mg/kg in milk, cheese, yogurt, custards and smoothie, butter, and soybean products [18]. Similarly, samples examined for Hg were below permissible limits except dried thyme (0.115 mg/kg) and thyme-flavored tea (0.105 mg/kg). Mercury was detected in all samples while 92% (33/36), and 78% (28/36) of the products were accepted according to LIBNOR and Codex Alimentarius, respectively. The average concentration was 0.076 mg/kg and ranged between 0.003 and 0.119 mg/kg. Remarkably, many of the samples tested for Pb exceeded the permissible limits (0.2–0.3 mg/kg) and most alarming were the samples of dried thyme (0.568 mg/kg), thyme-flavored tea (0.623 mg/kg), fresh thyme herb (0.601 mg/kg), and mixed thyme normal (0.791 mg/kg). Lead was detected in all the samples but only 14% (5/36) and 3% (1/36) were acceptable according to Libnor and Codex Alimentarius standards, respectively (Table S2). The average Pb concentration was 0.55 mg/kg and ranged between 0.014 and 0.876 mg/kg.

The average concentrations of Pb in dry ground thyme leaves (*Origanum syriacum)* (5.67 mg/kg) and in thyme mixture (*Origanum*, sumac, sesame, and salt) (8.55 mg/kg) previously collected from several stores in Beirut, Lebanon, showed much higher contamination levels than the same products assessed in our study. This variability can be related to several factors such as harvesting location sites, soil contamination, irrigation water and of environmental pollutions. Thus, there was a positive correlation between the presence of toxic metal Pb in soil and its level in the *Origanum syriacum* tissues, which in turn is reflected in the products derived from the plant.

Accumulating research has implicated the existence of these toxic heavy metals in many food matrices beyond permissible limits [19], [20]. Dghaim et al. conducted a study on traditional herbs found in the UAE market and showed that the maximum level of Cd in oregano and thyme samples were 0.35 mg/kg and 0.63 mg/kg, respectively, thus exceeding the permissible limit of cadmium 0.3 mg/kg set by the FAO/WHO [6]. Additionally, Pb exceeded the permissible limit and was present in average of 23.52 mg/kg in thyme samples [6]. Khozam et al. assessed the levels of toxic metals As, Cd, Hg and Pb in white cheese and indicated that As and Pb concentrations (0.022 mg/kg and 0.032 mg/kg respectively) were below the permissible limit set by the Codex Alimentarius for dairy products [21]. In a similar study, Bou Khouzam et al. determined the levels of toxic metals As, Cd, Hg and Pb in three different varieties of Lebanese bread (white, brown and Saj) from different geographical regions in wet and dry seasons. Mean concentrations in wet season (As 0.009 mg/kg, Cd 0.024 mg/kg, Pb 0.018 mg/kg) and in dry season (As 0.033 mg/kg, Cd 0.015 mg/kg, Pb 0.028 mg/kg) significantly varied between the two seasons and is attributed to the possibility of seasonal variation of wheat grain and water composition.

Many factors influence the toxicity of heavy metals in humans including sociodemographic factors such as age, sex, genes, and nutritional status in addition to the route of exposure, dosage, and chemical species. The heavy metals As, Cd, Hg and Pb occur naturally, are non-essential elements, non-biodegradable and their exposure can be increased by industrialization, urbanization, and anthropogenic activities. According to the substance priority list done by the Agency for Toxic Substances and Disease Registry (ATSDR), As, Pb, and Cd were ranked 1st, 2nd and 7th most potentially threatening substances to human health [22]. Heavy metals have an impact on cellular organelles and enzymes responsible of detoxifying, metabolizing and repairing damage in the body [23]. Due to their high toxicity, they can lead to multiple organ damage even at low concentrations and are categorized as human carcinogens according to the US Environmental Protection Agency and the International Agency for Research on Cancer [23].

Table 5 classifies each of these four heavy metals in terms of source, exposure, and health effects. Arsenic is a metalloid classified as a toxic metal widely used in the production of insecticides, herbicides, and growth stimulants for plants and animals. The main root of As exposure is via the consumption of contaminated foodstuffs and water. As exists in three forms: As, As^3+^ and As^5+^; each varying in toxicity to mammals according to organic or inorganic forms, physical state, and other factors such as solubility, particle size, cellular uptake, rates of absorption and elimination. As blocks cellular energy production and normal cell signaling, displaces other elements involved in fundamental chemical cellular processes and contributes to the development of diabetes, lung cancer and vascular disease [24].

**Table 5:** Exposure and health effects of As, Cd, Hg and Pb.

	**Source**	**Exposure**	**Health Effects**	**References**
As	volcanic eruptions, mining, burning of coal, pesticides, drugs, preservatives	Ingestion, inhalation, and skin absorption	skin lesions, circulatory disorders, pulmonary disorders, neurological complications, diabetes, hepatic and renal dysfunction, organ damage, malignant	[33], [34]
Cd	fertilizers, electronic wastes, industrial emission, mining and metal refining, batteries	Inhalation of fumes, ingestion of contaminated crops	kidney damage, cardiovascular disease, lung and gastrointestinal cancer,	[35], [36]
Hg	Coal power plants, gold and cement production, paper and pulp industry, medical waste, combustion of fossil fuels	Inhalation of fumes, ingestion of contaminated crops	Nervous system damage, immune and renal cancer	[37], [38]
Pb	mining, lead-based paints, agricultural practices, food packaging, pesticides	Inhalation of fumes, ingestion of contaminated crops	behavioral abnormalities, hearing deficits, neuromuscular weakness, impaired cognitive functions, toxicity in kidney and endocrine systems	[39], [40]

Cadmium is classified as carcinogenic to humans. In nature and in the environment, cadmium is known to occur in its inorganic form. All of the compounds related to cadmium are highly toxic to humans with dietary exposure being the most important pathway of exposure to the general population [25]. According to the European commission, the main source of Cd exposure is through the food chain more specifically cereal-based products, vegetables in addition to nuts and pulses [26]. Cd could reach the food chain due to many human activities like the use of chemical, fertilizers, pesticides, herbicides and animal manure in addition to the proximity of soil to industrial areas [27]. Adverse health issues include severe pain in joints and spine, kidney damage, hypertension and genetic mutations [28].

Mercury is one of the most toxic elements and is correlated with its chemical form. Mercury exists in three forms: elemental (Hg), inorganic (Hg^+^ and Hg^2+^) and organic (CH_3_Mg). Of all three, organic mercury is mostly toxic in terms of bioaccumulation and risk [29] and is found mostly in seafood while inorganic mercury is more common in fruits and vegetables [30]. Methylmercury is the most prevalent form of organic mercury found in the food chain. Effects of mercury toxicity lead to negative and harmful impact on several organs including the lungs, kidney, digestive system and immunity. The presence of Hg in food products is related to several reasons like the animal consumption of contaminated feed, the contamination of pasturelands with Hg-containing pesticides and fungicides [31]. This contamination can emerge from contaminated soils, irrigation water and deposits from polluted air. The source of contamination results from the combustion of fossil fuels, pulp and paper industries, medicinal waste products, production of cement and power plants.

The presence of Pb (elemental Pb and inorganic Pb^2+^) in thyme may result from two sources of contamination, one natural related to soil, and another resulting from human activities related to pesticides, painting, and industrial processes. The presence of Pb in the environment (air, soil and water) could be associated with anthropogenic activities and products such as industrial activities agrochemicals, oil-processing activities, water from old pipes, plumbing materials and natural events such as volcanic eruptions, mining areas and geochemical weathering. The source of Pb contamination in foodstuff is due to contamination during food preparation [9]. Pb is not metabolized by the human body but is rather absorbed directly into the bloodstream where fractions remain in the bones and teeth after replacing calcium, thus resulting in calcium deficiency and consequently osteoporosis. Pb also affects the peripheral and central nervous system, kidney function, and the metabolism of vitamin D. The International Agency for Research on Cancer IARC classified Pb as a carcinogen [32]. High levels of Pb exposure can have detrimental effects on vulnerable populations such as pregnant women leading to miscarriages [28]. The organic form of Pb is more readily absorbed by the gastrointestinal tract than the inorganic form, but most lead that is found in the environment is in the inorganic form.

## 4 Conclusion

We have assessed the levels of the toxic heavy metals As, Cd, Hg and Pb in thyme products by investigating their occurrences in various foodstuffs using microwave-assisted digestion and ICP-MS elemental analysis. Results showed that certain toxic metal concentrations were higher than permissible limits and can accumulate in the food chain. Some of the preventive and mitigation processes to decrease heavy metal content include careful monitoring and regulation of the use of fertilizers that contain heavy metals, proper management of waste products from industries and mining areas, and alternatives to the use of contaminated irrigation water and wastewater.

Further studies are required to assess the exposure and estimate the risk from consumption of these products. In addition, as the biotoxicity and carcinogenicity of toxic elements depend on their chemical form (organic or inorganic), speciation analysis could provide more accurate information on the form of toxic elements present to characterize with higher precision the health risk of consumers. The findings of our study highlight the need for planning best agricultural practices, implementation of a rigid food safety system, routine monitoring and update of regulations and standards related to toxic metals in food.

## Funding

This work was funded by Qatar National Library.

## CRediT authorship contribution statement

Caline Baroud collected samples, Sally El Kantar and Caline Baroud performed microwave digestion and contributed to manuscript writing, Elias Akoury and Sally El Kantar performed ICP-MS measurements, Hussein Hassan analyzed the data and wrote the manuscript, Elias Akoury and Layal Karam designed the study, analyzed the data, and wrote the manuscript.

## Declaration of Competing Interest

The authors declare that they have no known competing financial interests or personal relationships that could have appeared to influence the work reported in this paper.

## Data Availability

Data will be made available on request.
