# Supplementary material for: Determination of heavy metals contamination in thyme products by inductively coupled plasma mass spectrometry
Source: Toxicol Rep. 2022 Oct 27;9:1962–7. doi: 10.1016/j.toxrep.2022.10.014 (PMC9742941; doi:10.1016/j.toxrep.2022.10.014)

Determination of Heavy Metals Contamination in Thyme Products by Inductively Coupled Plasma Mass Spectrometry

Supplementary Information

**Supplementary Table 1.** Maximum allowed limits in mg/kg of different heavy metals in foodstuffs according to local and international standards

|  | | **As** | | **Cd** | | | **Hg** | | | **Pb** | | |  |
| --- | --- | --- | --- | --- | --- | --- | --- | --- | --- | --- | --- | --- | --- |
| **Foodstuffs** | **Local** | | **International** | | **Local** | **International** | | **Local** | **International** | | **Local** | **International** | |
| Thyme pie | 0.5^a^ | | 0.2^d^ | | 0.2^e^ | 0.2^g^ | | 0.5^h^ | 0.1^i^ | | 0.2^f^ | 0.2^r^ | |
| Cheese and thyme pie | 0.5^a^ | | 0.2^d^ | | 0.2^e^ | 0.2^g^ | | 0.5^h^ | 0.1^i^ | | 0.2^f^ | 0.2^r^ | |
| Thyme regular mix sandwich | 0.5^a^ | | 0.2^d^ | | 0.2^e^ | 0.2^g^ | | 0.5^h^ | 0.1^i^ | | 0.2^f^ | 0.2^r^ | |
| Thyme mix with nuts and seeds sandwich | 0.5^a^ | | 0.2^d^ | | 0.2^e^ | 0.2^g^ | | 0.5^h^ | 0.1^i^ | | 0.2^f^ | 0.2^r^ | |
| Pizza and pasta with thyme sauces | 0.5^a^ | | 0.2^d^ | | 0.2^e^ | 0.2^g^ | | 0.5^h^ | 0.1^i^ | | 0.2^f^ | 0.2^r^ | |
| Bread sticks with thyme (crunchy) | 0.5^a^ | | 0.2^d^ | | 0.2^e^ | 0.2^g^ | | 0.5^h^ | 0.1^i^ | | 0.2^f^ | 0.2^r^ | |
| Sesame thick bread with thyme (soft) | 0.5^a^ | | 0.2^d^ | | 0.2^e^ | 0.2^g^ | | 0.5^h^ | 0.1^i^ | | 0.2^f^ | 0.2^r^ | |
| Crackers with thyme | 0.5^a^ | | 0.2^d^ | | 0.2^e^ | 0.2^g^ | | 0.5^h^ | 0.1^i^ | | 0.2^f^ | 0.2^r^ | |
| Toast/bread with thyme | 0.5^a^ | | 0.2^d^ | | 0.2^e^ | 0.2^g^ | | 0.5^h^ | 0.1^i^ | | 0.2^f^ | 0.2^r^ | |
| Thyme croissant | 0.5^a^ | | 0.2^d^ | | 0.2^e^ | 0.2^g^ | | 0.5^h^ | 0.1^i^ | | 0.2^f^ | 0.2^r^ | |
| Aged cheese with thyme | 0.5^a^ | | 0.2^d^ | | 0.2^b^ | 0.2^l^ | | 0.5^h^ | 0.1^i^ | | 0.2^j^ | 0.02^k^ | |
| Tea infusion | 0.05^s^ | | 0.01^n^ | | 0.01^s^ | 0.003^o^ | | 0.001^s^ | 0.001^q^ | | 0.05^s^ | 0.01^p^ | |
| Tea soaked | 0.05^s^ | | 0.01^n^ | | 0.01^s^ | 0.003^o^ | | 0.001^s^ | 0.001^q^ | | 0.05^s^ | 0.01^p^ | |
| Fresh thyme salad | 0.5^a^ | | 0.2^d^ | | 0.2^b^ | 0.2^l^ | | 0.5^h^ | 0.1^i^ | | 0.3^c^ | 0.3^m^ | |
| Mixed thyme normal | 0.5^a^ | | 0.2^d^ | | 0.2^b^ | 0.2^l^ | | 0.5^h^ | 0.1^i^ | | 0.3^c^ | 0.3^m^ | |
| Mixed thyme extra | 0.5^a^ | | 0.2^d^ | | 0.2^b^ | 0.2^l^ | | 0.5^h^ | 0.1^i^ | | 0.3^c^ | 0.3^m^ | |
| Dried thyme herb | 0.5^a^ | | 0.2^d^ | | 0.2^b^ | 0.2^l^ | | 0.5^h^ | 0.1^i^ | | 0.3^c^ | 0.3^m^ | |
| Tea thyme | 0.5^a^ | | 0.2^d^ | | 0.2^b^ | 0.2^l^ | | 0.5^h^ | 0.1^i^ | | 0.3^c^ | 0.3^m^ | |

1. **Arsenic** Thyme.NL 677:2017 Lebanese Standards Institution LIBNOR
2. **Cadmium** Thyme.NL 677:2017 Lebanese Standards Institution LIBNOR
3. **Lead** Thyme.NL 677:2017 Lebanese Standards Institution LIBNOR
4. **Arsenic** Rice polished Codex Alimentarius CXS 193-1995
5. **Cadmium** Lebanese bread. NL 240:2010 Lebanese Standards Institution LIBNOR
6. **Lead** Lebanese bread. NL 240:2010 Lebanese Standards Institution LIBNOR
7. **Cadmium** Wheat. Codex Alimentarius CXS 193-1995
8. **Mercury** Instant tea.NL 610:2002. Lebanese Standards Institution LIBNOR
9. **Mercury** Salt, food grade. Codex Alimentarius CXS 150-1985.
10. **Lead** Chanklish.NL 507:2009 Lebanese Standards Institution LIBNOR
11. **Lead** Milk and secondary milk products. Codex Alimentarius 2015
12. **Cadmium** Leafy vegetables. Codex Alimentarius CXS 193-1995
13. **Lead** Leafy vegetables. Codex Alimentarius CXS 193-1995
14. **Arsenic** Natural mineral waters CXS 108-1981
15. **Cadmium** Natural mineral waters CXS 108-1981
16. **Lead** Natural mineral waters CXS 108-1981
17. **Mercury** Natural mineral waters CXS 108-1981
18. **Lead** Cereal grains. Codex Alimentarius CXS 193-1995
19. **Arsenic, Cadmium, Lead, Mercury** Bottled drinking water. NL 162:1999. Lebanese Standards Institution LIBNOR

***Supplementary Table 2.*** *Percentage of acceptable and unacceptable toxic element content in thyme products and dried thyme herbs in first and second collection according to LIBNOR and Codex Alimentarius Standards*

| **National/ International standard** | **Heavy metals** | **As** | **Cd** | **Hg** | **Pb** |
| --- | --- | --- | --- | --- | --- |
| LIBNOR standards | % of acceptable heavy metal concentration (n/N)^a^ | 89% (32/36) | 100% (36/36) | 92% (33/36) | 14% (5/36) |
|  | % of unacceptable heavy metal concentration (n/N)^a^ | 11% (4/36) | 0% | 8% (3/36) | 86% (31/36) |
| Codex Alimentarius standard | % of acceptable heavy metal concentration (n/N)^a^ | 89% (32/36) | 100% (36/36) | 78% (28/36) | 3% (1/36) |
|  | % of unacceptable heavy metal concentration (n/N)^a^ | 11% (4/36) | - | 22% (8/36) | 97% (35/36) |

^a^n/N: Number of acceptable samples/total number of samples in both categories at first and second collection

**Supplementary Figure 1.** Calibration curves of the heavy metals (A) As, (B) Cd, (C) Pb and (D) Hg.


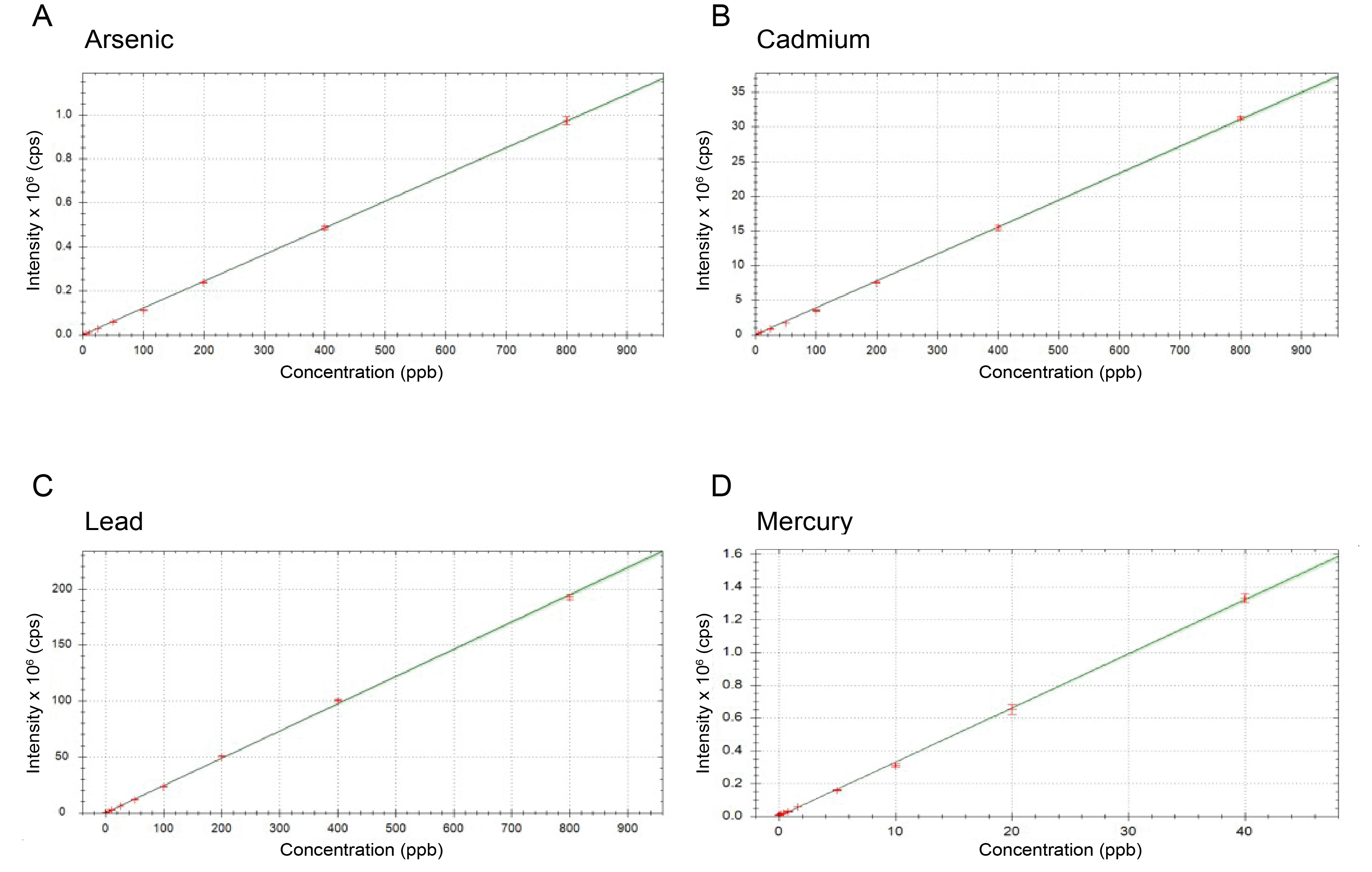

Supplement: Supplementary file 1 — Supplementary material. [file mmc1.docx]
